# Expression profiles and bioinformatic analysis of circular RNA in rheumatic heart disease: potential hsa_circ_0001490 and hsa_circ_0001296 as a diagnostic biomarker

**DOI:** 10.3389/fcvm.2025.1639767

**Published:** 2025-08-01

**Authors:** Xiaoliang Chen, Lina Chen, Li Bi, Shunying Zhao, Xiaoyan Hu, Ni Li, Linwen Zhu, Guofeng Shao

**Affiliations:** ^1^ Department of Cardiosurgery Intensive Care Unit, Ningbo Medical Centre Lihuili Hospital, Ningbo University, Ningbo, China; ^2^ Department of Cardiothoracic Surgery, Ningbo Medical Centre Lihuili Hospital, Ningbo University, Ningbo, China

**Keywords:** circular RNA, rheumatic heart diseases, regulatory network, diagnosis, biomarker

## Abstract

**Objective:**

Circular RNAs (circRNAs) are involved in various Cardiovascular diseases; however, the circRNA expression profiles and the circRNA-microRNA(miRNA)-messenger RNA (mRNA) regulatory network in rheumatic heart disease (RHD) remain poorly understood. This study aimed to investigate the expression profiles of circRNAs and construct a circRNA-miRNA-mRNA interaction network to reveal new diagnostic biomarkers and potential pathogenesis of RHD.

**Methods:**

Clinical data and plasma samples from 46 patients with RHD and 46 non-RHD patients were collected between January 2021 and December 2023. Arraystar Human CircRNA microarray was used to profile differentially expressed circRNAs in 3 paired samples (RHD vs. non-RHD). Quantitative real-time PCR (qRT-PCR) validated four candidate circRNAs in all 92 samples. The diagnostic value of differentially expressed circRNAs was analyzed by the Receiver Operating Characteristic (ROC) Curve. Bioinformatics analysis was used to predict the target miRNA and analyze the co-expressed mRNA to construct a circRNA–miRNA-mRNA regulatory network. The Gene Ontology (GO) and Kyoto Encyclopedia of Genes and Genomes (KEGG) pathway enrichment analyses were conducted to predict the potential functions of the differentially expressed genes and RHD-related pathways.

**Results:**

Four circRNAs were selected from circRNA microarray data. qRT-PCR confirmed that hsa_circ_0001490 and hsa_circ_0001296 were significantly upregulated in RHD plasma (4.28-fold, *P* < 0.001; 5.24-fold, *P* < 0.001, respectively). ROC analysis revealed hsa_circ_0001490 had an AUC of 0.792 (95% CI: 0.69–0.89; sensitivity: 93.5%; specificity: 67.4%), while hsa_circ_0001296 showed superior accuracy (AUC = 0.896; 95% CI: 0.83–0.96; sensitivity: 69.6%; specificity: 95.7%). A predicted hsa_circ_0001490-miRNA-mRNA regulatory network included 11 miRNAs and 1,973 mRNAs, and hsa_the circ_0001296-miRNA-mRNA interaction network included 9 miRNAs and 1,404 mRNAs. Moreover, the top 10 hub genes were screened within the two networks, respectively. The significantly enriched GO terms associated with hsa_circ_0001490 downstream genes were Smad binding and regulation of the Wnt signaling pathway. The significantly involved KEGG pathways included the Wnt signaling pathway, MAPK signaling pathway and TGF-beta signaling pathway. For hsa_circ_0001296, the significantly enriched GO terms were transforming growth factor beta receptor activity(type I) and Smad binding. The Autophagy pathway and MAPK signaling pathway were significantly involved in KEGG pathways.

**Conclusion:**

This study provides the first evidence of significant upregulation of hsa_circ_0001490 and hsa_circ_0001296 in RHD patients, suggesting their potential as diagnostic biomarkers for RHD. The constructed circRNA-miRNA-mRNA network reveals potential molecular mechanisms underlying RHD pathogenesis. Future studies should investigate these circRNAs' functional roles to fully elucidate their contribution to RHD development.

## Introduction

Rheumatic heart disease (RHD) is a post-infectious sequelae of acute rheumatic fever resulting from an abnormal immune response to streptococcal pharyngitis that triggers valvular damage, including stenosis and insufficiency and eventually develops into heart failure (1–3). Recent estimates suggest RHD affects approximately 40.5 million people worldwide (4, 5). Rheumatic heart disease represents a major global health burden, predominantly affecting young populations and causing significant cardiovascular morbidity and mortality (6). The current standard for rheumatic heart disease diagnosis remains based on clinical examination, the Jones criteria, and echocardiographic findings. However, the diagnosis criteria overly depend on clinicians' technical skills and judgment, resulting in poor sensitivity and specificity (7, 8). Novel biomarkers could significantly improve RHD diagnostic accuracy. CircRNAs show promise as potential diagnostic biomarkers that could establish new standards for RHD diagnosis (9).

Recent studies indicate that circRNA plays a crucial role in the occurrence and progression of cardiovascular diseases (10). Because circRNA is not easily degraded by exonuclease, its half-life is significantly longer than that of linear RNA. Its high abundance, relative stability, and evolutionary conservation in the peripheral blood distinguish it from traditional linear RNAs. Certain circRNAs show potential as biomarkers for their diagnosis (9). Currently, there are limited studies on the role and mechanisms of circRNA in RHD. This study aims to identify RHD-specific circRNAs and construct their regulatory networks, which may provide new insights into circRNA functions as potential diagnostic biomarkers and reveal underlying disease mechanisms.

## Materials and methods

### CircRNA microarray assays

In the present study, the expression profile of circRNA in the plasma of three RHD patients and three non-RHD patients was analyzed using the Arraystar Human CircRNA microarray. Differentially expressed circRNAs were identified and had been uploaded to the GEO database (GSE168932, https://www.ncbi.nlm.nih.gov/geo/query/acc.cgi?acc=GSE168932). Candidate circRNAs with fold-change >1.5 and *p* < 0.05 were selected. We conducted a literature search on circRNAs that had not been previously studied in RHD. Ultimately, we chose four circRNAs as our research subjects (Table 1).

**Table 1:** Basic information on differentially expressed circRNA in circRNA microarray assays.

CircRNA	*P*-value	logFC	Regulation	chrom	txStart	txEnd	Strand	Gene Symbol
hsa_circ_0001255	0.007534801	2.2390883	up	chr22	50,618,381	50,618,469	+	PANX2
hsa_circ_0001490	0.002455683	2.1015474	up	chr5	61,653,517	61,657,356	+	KIF2A
hsa_circ_0001296	0.025225604	1.9614110	up	chr3	47,719,687	47,727,660	−	SMARCC1
hsa_circ_0068655	0.031363089	1.8681526	up	chr3	196,129,822	196,134,264	−	UBXN7

logFC, log2 fold change; txStart, transcript start; txEnd, transcript end.

### Study population

Ninety-two plasma samples were obtained from the inpatients in the Department of Cardiothoracic Surgery at Ningbo Medical Centre Lihuili Hospital from January 2021 to December 2023. All patients were diagnosed with clinical symptoms, physical examination, electrocardiograph (ECG), chest x-ray, and TTE. The RHD group met the following criteria: (1) age ≥18 years old. (2) meeting the RHD diagnostic criteria (7), (3) at least one of the heart valves is damaged. The non-RHD group was recruited during the same period from the Department of Cardiothoracic Surgery at Ningbo Medical Centre Lihuili Hospital. They were diagnosed with non-rheumatic heart valve disease. All patients meeting the exclusion criteria were: (1) patients with valvular heart disease combined with coronary heart disease or acute myocardial infarction. (2) patients with infective endocarditis. A propensity score match was used to pair patients between the RHD and non-RHD groups. This study was approved by the Medical Ethics Committee of Ningbo Medical Center Li Huili Hospital (Approval No. 2023310). Written informed consent was obtained from all participants.

### Collection and storage of plasma samples

Peripheral blood samples were collected into EDTA-containing anticoagulation tubes, and plasma was separated within a maximum of 3 h of blood withdrawal by centrifugation of the tubes at 3,000 rpm for 15 min at 4°C. The upper plasma layer was then removed into 2 ml RNase-free Eppendorf tubes and stored at −80°C.

### Total RNA extraction and quantitative real-time PCR

According to the manufacturer's instructions, total RNA was extracted from plasma using TRIzol LS reagent (Invitrogen). RNA purity was assessed by measuring A260/A280 ratios. The integrity of the RNA was assessed by denaturing agarose gel electrophoresis. cDNA was synthesized using the GoScript Reverse Transcription System (Promega, USA) with gene-specific primers. We used the Applied Biosystems 7,500 Realtime PCR system (ThermoFisher Scientific, USA) to amplify four circRNAs using GoTaq qPCR Master Mix (Promega, USA) according to the kit instructions. Glyceraldehyde-3-phosphate dehydrogenase (GAPDH) mRNA served as the endogenous control for relative quantification. The primer sequences were designed in the laboratory and synthesized by Genepharma Biotech (Genepharma, China) based on the mRNA sequences obtained from the National Center for Biotechnology Information database. BLAST was used to verify the specificity of the PCR primers. The specific convergent primer sequences of four circRNAs and GAPDH primer sequences are shown in Table 2. Amplification curve and melting curve analysis were performed to validate the specific generation of the expected PCR product. The expression fold change of the circRNAs was then calculated using the 2^−ΔΔCt^ method.

**Table 2:** Primers designed for quantitative real-time PCR validation of selected circRNAs.

Gene symbol	Forward primer	Reverse primer	Product length (bp)
hsa_circ_0001255	TGCCAGCTTGAGTGGGAGT	ACACGTGCACACTCGTACAT	65
hsa_circ_0001490	GGGCAGACTGGAAGTGGAAA	CCTATGTTCATCAATCTGCTAATGC	106
hsa_circ_0001296	GATTGACACGTATCGTCTAAACCC	TGGCAGGATCTTCATCTTCCTC	109
hsa_circ_0068655	AAGAAGAAGTTCGTGCCCCA	TACACTTTCACTTGCACCACCAA	86
GAPDH	GATGAGAAGTATGACAACAGCCT	AGTCCTTCCACGATACCAAAGT	113

GAPHD, glyceraldehyde-3-phosphate dehydrogenase.

### Prediction of target miRNAs for circRNAs and target mRNAs for miRNAs

To predict the interactions between circular circRNAs and miRNAs, we utilized several online databases, including circAtlas 3.0 (http://circatlas.biols.ac.cn), Circular RNA Interactome (https://circinteractome.nia.nih.gov/mirna_target_sites.html), and circBank (https://www.circbank.cn/#/home). The miRNAs predicted simultaneously by the circAtlas, the Circular RNA Interactome, and circBank were considered target miRNAs of the circRNAs. For identifying the downstream target mRNAs of these miRNAs, we employed the online databases TargetScanHuman 8.0 (http://www.targetscan.org/), DIANA-microT (http://diana.imis.athena-innovation.gr/DianaTools/index.php), and miRDB (https://mirdb.org/index.html). Similarly, the mRNAs predicted by TargetScan, DIANA-microT, and miRDB were classified as candidate downstream target mRNAs simultaneously. A Venn diagram was used to improve predictive accuracies (via intersection) (http://bioinformatics.psb.ugent.be/webtools/Venn/). Using the information from circRNAs, miRNAs, and mRNAs, we constructed a circRNA-miRNA-mRNA network.

### GO and KEGG pathway enrichment analyses

The GO and KEGG pathway enrichment analyses were applied to investigate the potential functions of differentially expressed circRNAs. The GO and KEGG analyses were performed to identify the downstream target genes of miRNAs, which were completed using the Metascape online software (https://metascape.org/gp/index.html). GO analysis was applied to annotate the genes with terms under the biological process (BP), cellular component (CC), and molecular function (MF) categories. KEGG pathway analysis was performed to identify the significant pathways associated with the differentially expressed genes.

### Construct the protein-protein interaction network of target genes

The protein interactions for the target genes were obtained from the STRING database (https://string-db.org/), using a minimum required interaction score ≥0.9 as the threshold. A protein-protein interaction network(PPI) was then constructed using Cytoscape version 3.10.3 software. The top ten genes were identified as hub genes through the MCC algorithm in the CytoHubba plugin.

### Construction of the circRNA-miRNA-mRNA regulatory network

The top 10 hub genes screened were further constructed by the circRNA-miRNA-mRNA regulatory network, which was visualized using Cytoscape software (version 3.10.3).

### Statistical analysis

For numerical variables, a normal distribution was assessed using the Kolmogorov–Smirnov test. Numerical variables with a normal distribution are expressed as mean ± standard deviation (SD) and compared using Student's *t*-test; otherwise, they are expressed as median (Q25, Q75) and compared using the Mann–Whitney *U* test. Categorical data are shown as numbers (%) and analyzed using the chi-square test or Fisher's exact test as appropriate. The diagnostic value of circRNAs was analyzed by receiver operating characteristic (ROC) curves and the area under the ROC curve (AUC). A two-tailed *p*-value < 0.05 was considered to indicate a significant difference. All statistical analyses were performed using SPSS Statistics 26.0 (IBM Corp., Armonk, NY) and GraphPad Prism 9.0 (GraphPad Software, USA) software.

## Results

### Clinical characteristics comparison between the RHD group and the non-RHD group

In the 92 patients, there were no significant differences in clinical characteristics between the RHD and control groups (*P* > 0.05) (Table 3).

**Table 3:** Clinical characteristics of patients used for validation between the two groups.

Valuables	RHD group (*n* = 46)	non-RHD group (*n* = 46)	*p* Value
Age, years	64.39 ± 10.53	64.30 ± 13.09	0.972
Female, *n* (%)	22 (47.83%)	21 (45.65%)	0.834
BMI (kg/m^2^)	23.27 ± 3.77	22.24 ± 3.34	0.167
C-reactive protein (mg/L)	1.64 (0.81,10.86)	2.36 (0.62,7.96)	0.863
Hemoglobin (g/L)	11.92 ± 2.23	12.63 ± 2.07	0.116
Hematocrit (%)	36.56 ± 6.34	37.68 ± 5.59	0.372
Albumin (g/L)	36.44 ± 4.76	38.09 ± 3.99	0.074
Creatinine (μmol/L)	77.50 (60.00, 121.25)	70.00 (58.00, 92.25)	0.108
NYHA class III–IV, *n* (%)	40 (86.96%)	39 (84.78%)	0.765
Hypertension, *n* (%)	16 (34.78%)	19 (41.30%)	0.519
Atrial fibrillation, *n* (%)	22 (47.83%)	16 (34.78%)	0.204
Diabetes mellitus, *n* (%)	6 (13.04%)	2 (4.35%)	0.267
Pulmonary hypertension, *n* (%)	11 (23.91%)	8 (17.19%)	0.440

RHD, rheumatic heart diseases.

### Plasma circRNA relative expression

**Figure 1:**
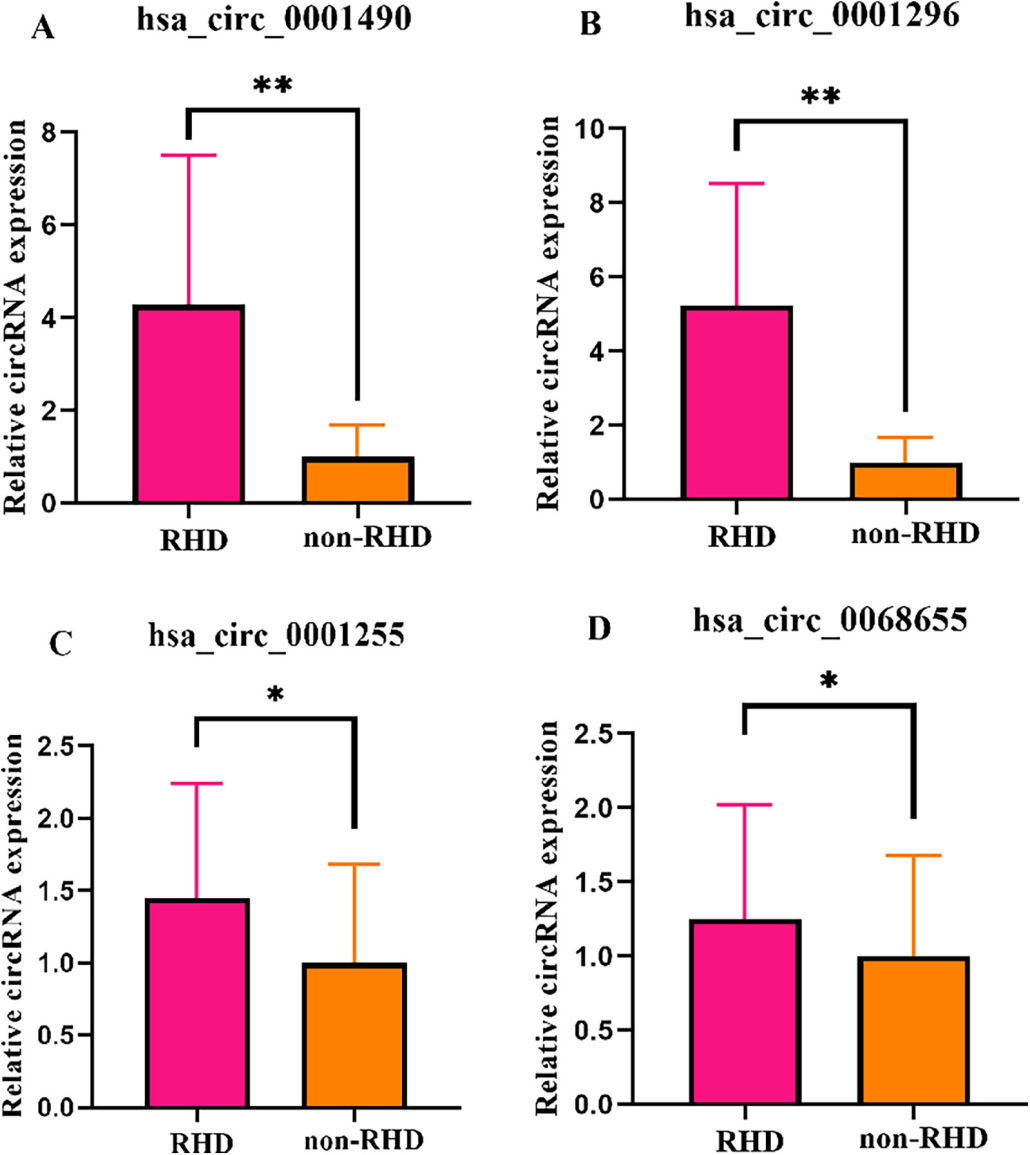
The expression levels of circRNAs were validated in both the RHD and non-RHD groups using qRT-PCR. Relative plasma expression (fold change) of hsa_circ_0001490 **(A)**, hsa_circ_0001296 **(B)**, hsa_circ_0001255 **(C)** and hsa_circ_0068655 **(D)** in patients with RHD compared to non-RHD patients. *p > 0.05; **p < 0.01.

Four candidate circRNAs were validated in a large sample using qRT-PCR, and the results were confirmed through agarose gel electrophoresis. Hsa_circ_0001490, hsa_circ_0001296, hsa_circ_0001255, and hsa_circ_0068566 expression fold change in plasma samples of patients with RHD calculated relative to non-RHD patients is shown in Figure 1. Plasma hsa_circ_0001490 and hsa_circ_0001296 relative expression were significantly elevated in patients with RHD (3.94 ± 3.50) (*P* < 0.001) and (2.70 ± 1.99) (*P* < 0.001), respectively (Figures 1A,B). However, there were no significant differences in the relative expression of plasma hsa_circ_0001255 (*P* = 0.051) and hsa_circ_0068655 (*P* = 0.073) between the two patient groups (Figures 1C,D).

### Diagnostic efficacy of hsa_circ_0001490 and hsa_circ_0001296 for RHD

**Figure 2:**
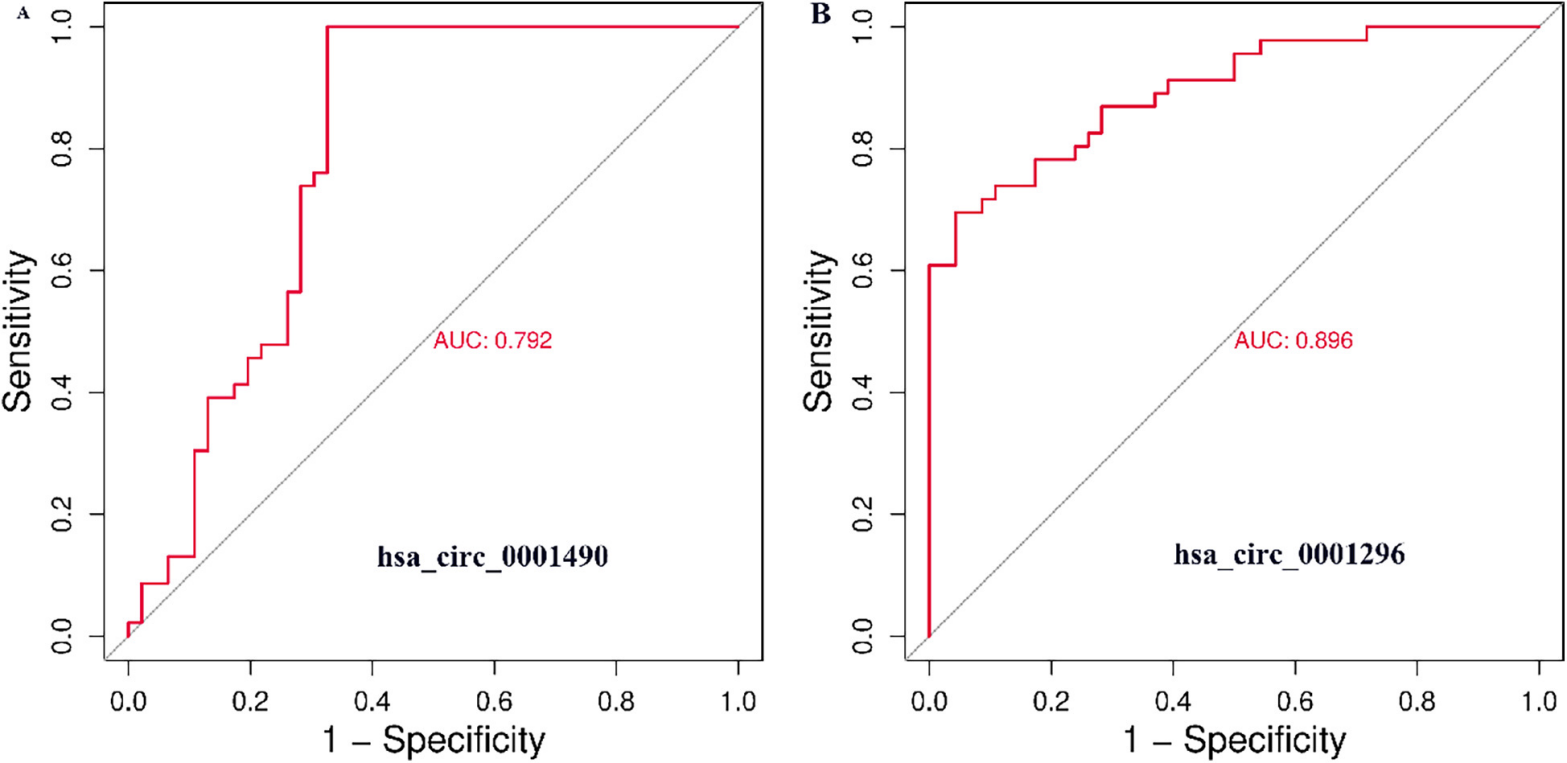
ROC curve analysis of plasma hsa_circ_0001490 (AUC = 0.792, cutoff point = 1.47, *P* < 0.001, sensitivity = 93.5% and specificity = 67.4%) **(A)** and hsa_circ_00011296 (AUC = 0.896, cutoff point = 1.69, *P* < 0.001, sensitivity = 69.6% and specificity = 95.7%) **(B)** were performed to predict RHD.

The diagnostic potential of plasma hsa_circ_0001490 and hsa_circ_0001296 expression fold change was assessed by plotting ROC curves for RHD status (Figures 2A,B). The area under the curve (AUC) for hsa_circ_0001490 was 0.792 (95% CI, 0.69–0.89, *P* < 0.001), and the cutoff value for hsa_circ_0001490 fold change for predicting RHD was 1.47 (sensitivity = 93.5%, specificity = 67.4%). Similarly, the AUC for hsa_circ_0001296 was 0.896 (95% CI, 0.83–0.96, *P* < 0.001) for RHD, and the threshold for the hsa_circ_0001296 fold change in predicting RHD was 1.69 (sensitivity, 69.6%; specificity, 95.7%).

### Prediction of circRNA-miRNA-mRNA regulatory network

Bioinformatic predictions were performed for two significantly differentially expressed circRNAs to construct their circRNA-miRNA-mRNA Regulatory networks. For hsa_circ_0001490, potential miRNA binding sites were predicted using three databases (circAtlas, the Circular RNA Interactome, and circBank). This analysis identified 11 high-confidence miRNAs with conserved binding sites across all three databases. Downstream targets of these miRNAs were predicted using TargetScan, DIANA-microT, and miRDB. Initial screening yielded 2,199 target mRNAs, which were refined to 1,973 non-redundant mRNAs after intersection analysis (Table 4 and Supplementary Figure S1). Similarly, for hsa_circ_0001296, the analysis identified 9 high-confidence miRNAs with conserved binding sites, and the initial screening yielded 1,468 target mRNAs, which were refined to 1,404 non-redundant mRNAs after intersection analysis (Table 4 and Supplementary Figure S2).

**Table 4:** Predicted results of circRNAs-miRNAs and miRNAs-mRNAs.

circRNA	microRNA	Intersected genes
	hsa-miR-145-5p	77
	hsa-miR-330-3p	339
	hsa-miR-346	48
	hsa-miR-329-5p	66
	hsa-miR-622	203
hsa_circ_0001490	hsa-miR-659-3p	332
	hsa-miR-937-5p	89
	hsa-miR-3978	437
	hsa-miR-4436b-5p	59
	hsa-miR-5003-5p	430
	hsa-miR-8077	119
	hsa-miR-509-3p	154
	hsa-miR-510-5p	233
	hsa-miR-3192-3p	33
	hsa-miR-4326	222
hsa_circ_0001296	hsa-miR-3925-3p	251
	hsa-miR-4431	5
	hsa-miR-4714-5p	165
	hsa-miR-6773-3p	187
	hsa-miR-8070	218

### GO and KEGG pathway analysis of circRNAs downstream genes

**Figure 3:**
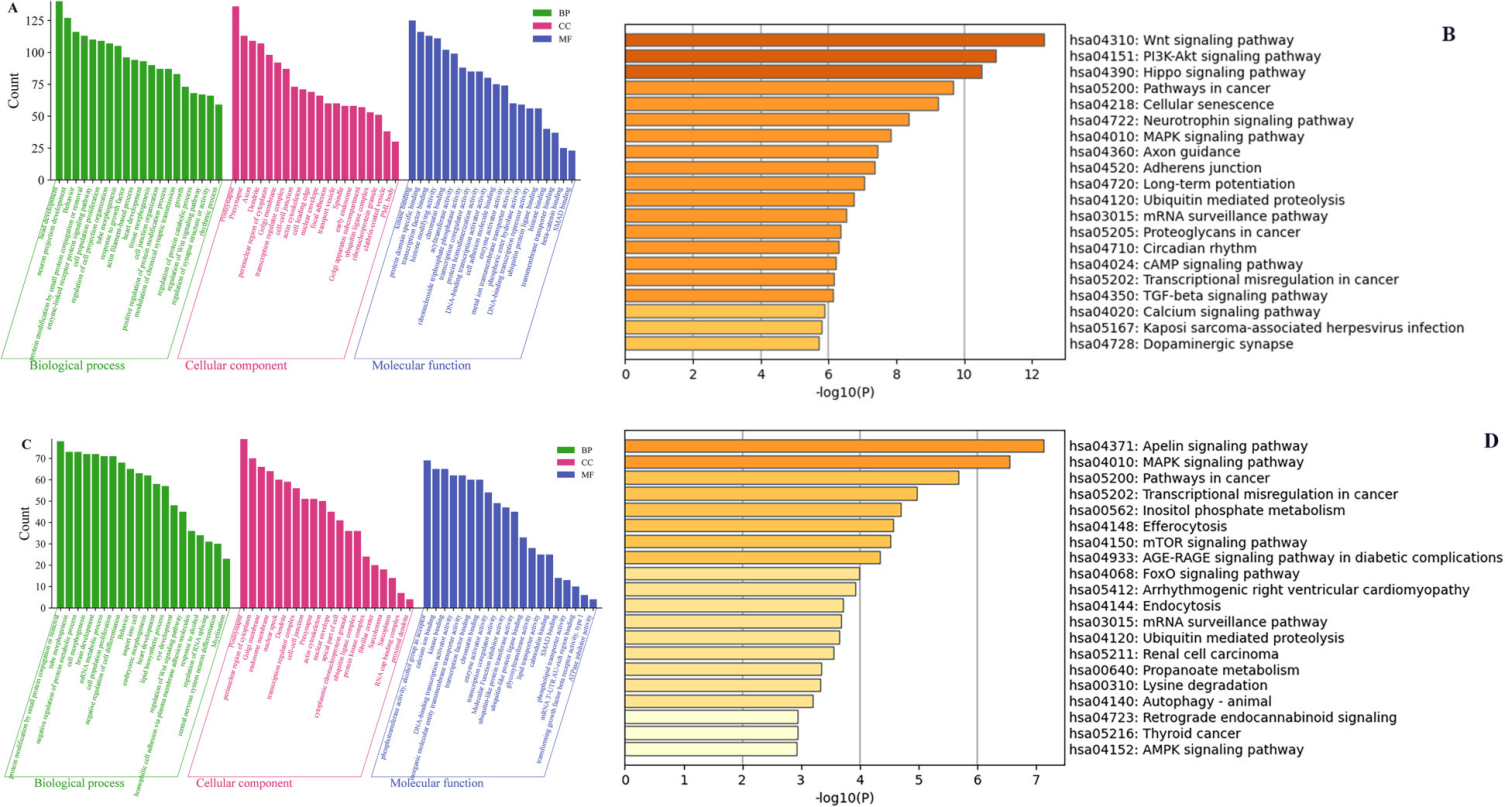
The GO and KEGG pathway enrichment analyses of the downstream target genes for differentially expressed circRNAs. The top 20 significantly enriched BP, CC, and MF terms by GO analysis **(A)** and the top 20 significantly enriched pathways by KEGG analysis **(B)** of the downstream genes of hsa_circ_0001490. The top 20 significantly enriched BP, CC, and MF terms by GO analysis **(C)** and the top 20 significantly enriched pathways by KEGG analysis **(D)** of the downstream genes of hsa_circ_0001296. GO, Gene Ontology; KEGG, Kyoto Encyclopedia of Genes and Genomes; BP, biological process; CC, cellular component; MF, molecular function. Column length represents enrichment score (−log10[*P*-value]), with longer bars indicating greater statistical significance.

We conducted the GO and KEGG pathway analyses to predict the potential functions of circRNAs. The predicted functional terms with *p*-value < 0.05 were selected and ranked by enrichment score [−log10 (*p*-value)]. For hsa_circ_0001490 targets, the top 20 enriched GO terms spanned biological processes (BP), cellular components (CC), and molecular functions (MF) (Figure 3A). KEGG analysis revealed the top 20 potentially relevant pathways for RHD pathogenesis (Figure 3B). Similarly, hsa_circ_0001296 targets showed enrichment in 20 top GO terms across BP, CC, and MF categories (Figure 3C). Corresponding KEGG analysis identified 20 top pathways potentially associated with RHD (Figure 3D).

### Protein-protein interaction network analysis and hub genes screening

**Figure 4:**
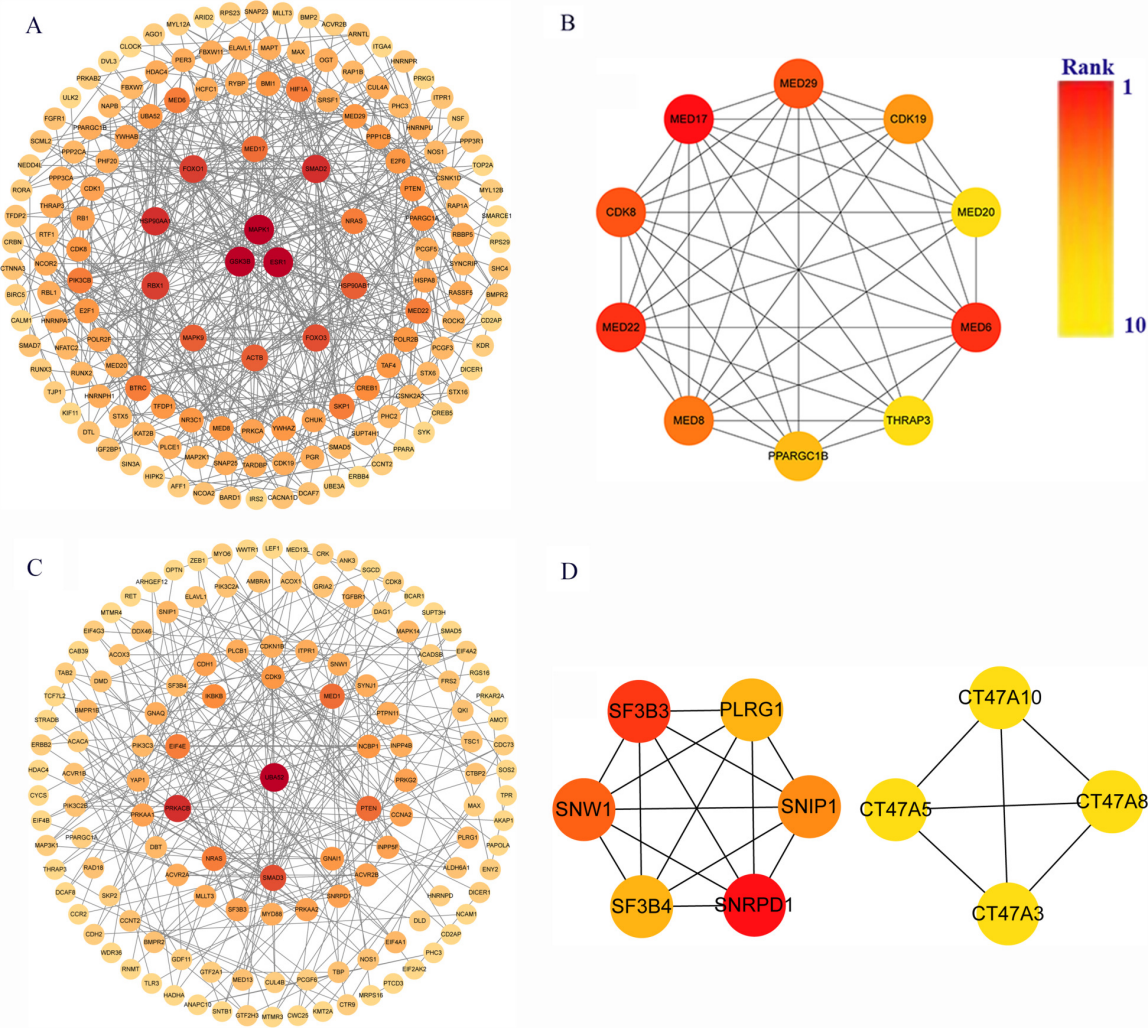
Identification of key genes in rheumatic heart disease pathogenesis. **(A)** Protein-protein interaction (PPI) network of 1,973 hsa_circ_0001490 downstream targets in the ceRNA network. **(B)** Top 10 hub genes from hsa_circ_0001490's PPI network. **(C)** PPI network of 1,404 hsa_circ_0001296 downstream targets in the ceRNA network. **(D)** Top 10 hub genes from hsa_circ_0001296's PPI network. PPI, Protein-protein interaction. Circular nodes represent genes; node darkness corresponds to degree score, reflecting interaction strength.

The target genes of miRNAs associated with hsa_circ_0001490 and hsa_circ_0001296 were analyzed for functional interactions using the STRING database. The resulting interaction networks were visualized and analyzed using Cytoscape (Figures 4A,C). The significant hub genes were identified using the CytoHubba plugin with the Molecular Complex Detection (MCC) method. The top ten hub genes for each circRNA were as follows: For the hsa*circ*0001490 network, these were MED17, MED22, MED6, MED29, CDK8, MED8, CDK19, PPARGC1B, MED20, and THRAP3. For the hsa_circ_0001296 network: SNRPD1, SF3B3, SNW1, SNIP1, PLRG1, SF3B4, CT47A10, CT47A5, CT47A3, and CT47A8 (Figures 4B,D).

### Construction of the circRNA–miRNA-hub gene network

**Figure 5:**
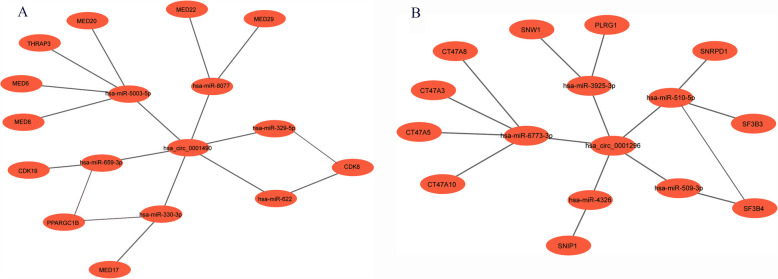
The circRNA-miRNA-mRNA regulatory network. (A) The hsa_circ_0001490-miRNA-hub gene regulatory network: In this network, hsa_circ_0001490 could affect functions of 6 miRNAs and then regulate the 10 downstream hub genes. (B) The hsa_circ_0001296-miRNA-hub gene regulatory network: In this network, hsa_circ_0001296 affects the functions of 5 miRNAs, leading to the regulation of 10 downstream hub genes.

Based on bioinformatic predictions, hsa_circ_0001490 interacted with six miRNAs (hsa-miR-5003-5p, hsa-miR-659-3p, hsa-miR-330-3p, hsa-miR-8077, hsa-miR-622, and hsa-miR-329-5p) and ten hub genes, while hsa_circ_0001296 was associated with five miRNAs (hsa-miR-510-5p, hsa-miR-3925-3p, hsa-miR-6773-3p, hsa-miR-4326, and hsa-miR-509-3p) and an additional ten hub genes. These interactions formed a comprehensive circRNA–miRNA–hub gene regulatory network, suggesting a potential competing endogenous RNA (ceRNA) mechanism in RHD (Figures 5A,B).

## Discussion

Rheumatic heart disease is responsible for nearly 250,000 deaths annually and poses a significant health threat in developing areas. However, the molecular mechanisms underlying RHD remains poorly understood, and there is a lack of specific diagnostic methods (7, 11). Although valvular surgery serves as the primary treatment, its high costs and requirement for long-term postoperative medication create substantial socioeconomic burdens for patients in developing areas (12, 13). These limitations highlight the urgent need for novel diagnostic biomarkers and improved therapeutic strategies to enable early detection and targeted interventions. CircRNA is highly conservative, and increasing studies show that it plays an important role in the diagnosis and pathogenesis of Cardiovascular diseases (14). Previous studies assessed circRNA as a potential biomarker for coronary heart disease (15), acute myocardial infarction (16), and heart failure (17). For instance, Xiong et al. elucidated the circRNA-miRNA-mRNA network in sepsis-induced cardiovascular dysfunction (18). However, there are relatively few studies on circRNA in RHD.

Our study revealed significant upregulation of hsa_circ_0001490 and hsa_circ_0001296 in RHD patients' plasma, with ROC analysis supporting their potential as diagnostic biomarkers (AUC = 0.792 and 0.896, respectively). In our previous studies, the researchers selected 42 patients RHD who had severe valvular lesions necessitating valve surgery, alongside 42 non-RHD patients and 42 healthy individuals. The findings indicated that hsa_circ_0000437 was elevated in surgical RHD cases compared to controls (19). However, that study's cohort was limited to severe valvular lesions, whereas our current study included all RHD patients meeting clinical criteria (7), enhancing generalizability. Comparatively, Hu et al. (20) conducted a study on nine samples of atrial tissue from patients with atrial fibrillation combined with RHD. The researchers identified six dysregulated circRNAs ((circRNA_20118, circRNA_17558, circRNA_16688, circRNA_11109, circRNA_11017, and circRNA_11058) in atrial tissue from RHD patients with atrial fibrillation. However, they focused only on patients with RHD combined with atrial fibrillation, collecting atrial tissue samples proved difficult, and a small sample size limited clinical applicability. In contrast, our use of peripheral blood offers practical advantages for broader diagnostic implementation.

Rheumatic heart disease mainly manifests as valvular damage caused by recurrent rheumatic fever, such as valve fibrosis, stenosis and calcification (21). hsa_circ_0001490 is a circular RNA located on chromosome 5(chr5: 61653517-61657356), which is transcribed and reverse-spliced from the KIF2A gene. We predicted the potential functions of the differentially expressed hsa_circ_0001490 using the GO and KEGG pathway analyses in patients with RHD. GO analysis revealed that hsa_circ_0001490's downstream target genes regulate multiple biological processes, such as Smad binding, regulation of the Wnt signaling pathway, transcription factor binding, and response to growth factor. The endothelial-mesenchymal transition (EndMT) plays a key role in several diseases, including cardiac fibrosis. Smads are key regulators of EndMT, as their phosphorylation (Smad2/3) modulates EndMT-related transcription factors, suggesting EndMT involvement in RHD (22). KEGG analysis indicated that the target genes were involved in the Wnt signaling pathway, TGF-beta signaling pathway, and MAPK signaling pathway. Guo and colleagues demonstrated that the Wnt signaling pathway may be involved in the increased expression of Snail1 and atrial fibrosis in patients with atrial fibrillation and RHD. These findings suggested the Wnt signaling pathway promoted EndMT development, potentially enhancing Snail1 levels and atrial fibrosis in AF and RHD patients (23). The transforming growth factor-beta(TGF-β) signaling pathway is a major regulator of EndMT, with key factors such as activin, Smad2, and Smad3 (24). Xiao et al. demonstrated that the TGF-β1/Smad signaling pathway is involved in the RHD-mediated atrial fibrosis (25). Cardiac fibrosis is promoted through the activation of MAPK and their downstream signaling, including their interaction with TGF-β and Smad proteins. Cardiac fibrosis further causes calcification and stiffening of the heart valves in RHD (26).

CircRNAs primarily function as potent miRNA sponges, modulating the biological activity of downstream miRNAs and their target mRNAs in cardiovascular diseases (27, 28). We predicted a potential hsa_circ_0001490-miRNA-mRNA regulatory network that may be involved in the pathogenesis of RHD using bioinformatics tools. We further constructed a protein-protein interaction network for the target genes and identified the top 10 hub genes: MED17, MED22, MED6, MED29, CDK8, MED8, CDK19, PPARGC1B, MED20, and THRAP3. The Mediator complex (MED) is a vital multiprotein transcriptional co-regulator that plays a crucial role in activating the transcription machinery during the initial stages of RNA polymerase II recruitment, as well as the subsequent processing and termination steps of transcription. Increasing evidence suggests that MED is involved in major cardiovascular diseases (29). Cyclin-dependent kinases 8 (CDK8) and 19 assemble with cyclin C, Med12, and Med13 to form the Mediator kinase module, which is reversibly associated with MED, controlling gene transcription both positively and negatively. The researchers found that pharmacological inhibition of CDK8 and CDK19, which were reversibly associated with MED and mainly control the function of transcription factors positively and negatively, was able to induce the transcription factor forkhead box protein 3(Foxp3) expression in naïve and effector/memory T cells, thereby suppressing harmful immune responses (30). The abnormally expressed hsa_circ_0001490 in RHD patients may affect the occurrence and development of RHD by regulating the expression of related downstream target genes.

Hsa_circ_0001296 is formed by the reverse splicing of exons 14, 15, and 16 of the gene SMARCC1 located on chromosome 3 (chr3: 47719687-47727660). Its expression was significantly upregulated in RHD patients, suggesting its potential as a diagnostic biomarker for RHD. However, the molecular mechanisms underlying its role in RHD pathogenesis remain unclear. Using GO and KEGG pathway analyses, we predicted the functional roles of hsa_circ_0001296 in RHD. GO analysis revealed that its downstream target genes regulate multiple biological processes, including transforming growth factor beta receptor activity (type I), transcription factor binding, regulation of Wnt signaling pathway, and Smad binding. TGF-β1, a key member of the TGF-β family and a major secretory cytokine of regulatory T cells (Tregs), induces phosphorylation of membrane-bound receptors to mediate nuclear signaling. Zhou et al. suggested that overexpressed TGF-β1 in valvular tissues had a potential role in the myofibroblast proliferation, valvular fibrosis, inflammatory cell infiltration and valve calcification, which might cause the scarring sequelae of RHD (31). KEGG analysis revealed that the Autophagy pathway and MAPK signaling pathway were closely associated with the downstream target genes of hsa_circ_0001296. Autophagy is a self-degradative pathway by which subcellular elements are broken down intracellularly to maintain cellular homeostasis (32). The PTEN-induced putative kinase protein 1 (PINK1)/Parkin pathway is a well-known autophagic pathway (33). Excessive autophagy has been linked to valve diseases, resulting in the recruitment of inflammatory cells and calcification (32, 34). Bai et al. revealed that IL-17 plays a crucial role in EndMT in RHD via the PINK1/Parkin autophagic pathway and macrophage polarization, providing a potential therapeutic target (21). Zhou, et al. preliminarily explored the mechanism of CD4^+^ T cells and the TGF-β1/MAPK pathway involved in the pathological process of valvular hyperplasia and fibrosis of RHD (31).

Furthermore, we predicted a potential hsa_circ_0001296-miRNA-mRNA regulatory network that may contribute to RHD pathogenesis. Protein-protein interaction network analysis identified the top 10 hub genes: SNRPD1, SF3B3, SNW1, SNIP1, PLRG1, SF3B4, CT47A10, CT47A5, CT47A3, and CT47A8. SNIP1 is a transcriptional repressor that inhibits the BMP signaling pathway by directly interacting with SMAD2/3 proteins, thereby limiting their effects. Furthermore, SNIP1 modulates TGF-β/BMP signaling by disrupting the association between SMAD2/3 and the histone acetyltransferases CBP/p300 (35). The nuclear factor-kappa B (NF-κB) family of transcription factors is central to regulating gene expression involved in inflammation, immune responses, cell proliferation, and various other biological processes. SNW1 is a novel regulator of the NF-κB pathway activation in human macrophages (36). These findings suggest that dysregulated hsa_circ_0001296 expression in RHD patients may influence disease progression through modulation of this circRNA-miRNA-mRNA network.

This study has several limitations. First, the sample size of the circRNA microarray assays in this study is still limited, which may increase the false-positive rate and reduce statistical power. We plan to further validate these findings in future experiments. Second, in this study, we constructed a circRNA-miRNA-mRNA regulatory network and analyzed hub genes through bioinformatics analysis of two circRNAs that exhibited significant expression differences in patients with RHD. However, these findings require further validation through *in vivo* and *in vitro* experiments. Third, we cannot exclude the possibility that hsa_circ_0001490 and hsa_circ_0001296 may be overexpressed in other types of cardiovascular diseases in our population, as we did not comprehensively include them in our study. Thus, the generalizability of these findings requires confirmation in larger, multicenter studies.

## Conclusions

We are reporting for the first time that hsa_circ_0001490 and hsa_circ_0001296 are highly expressed in the plasma of patients with RHD. We further constructed a circRNA-miRNA-target gene ceRNA regulatory network that is related to the pathogenesis of RHD. This work lays the groundwork for further exploration of the molecular mechanisms underlying RHD and the identification of potential circRNA diagnostic biomarkers for the disease.

## Data Availability

The original contributions presented in the study are included in the article/Supplementary Material, further inquiries can be directed to the corresponding author.
