# Supplementary figures and images for: Expression profiles and bioinformatic analysis of circular RNA in rheumatic heart disease: potential hsa_circ_0001490 and hsa_circ_0001296 as a diagnostic biomarker
Source: Front Cardiovasc Med. 2025 Aug 1;12:1639767. doi: 10.3389/fcvm.2025.1639767 (PMC12354471; doi:10.3389/fcvm.2025.1639767)

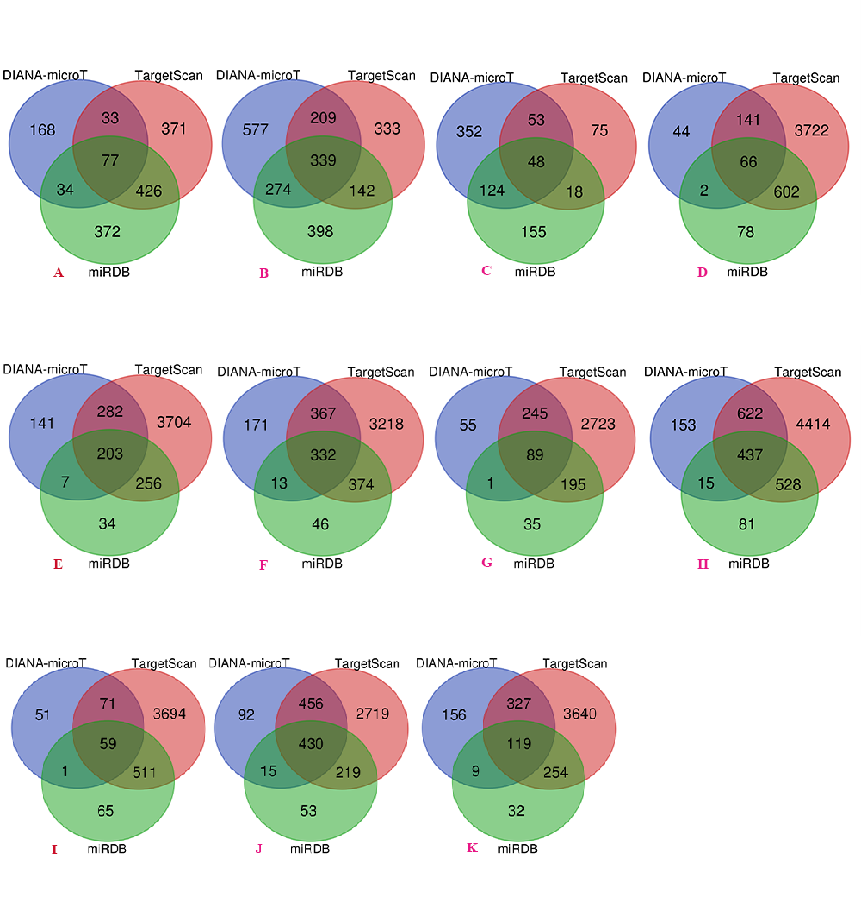

Supplement: Supplementary file 1 [file Image1.tif]

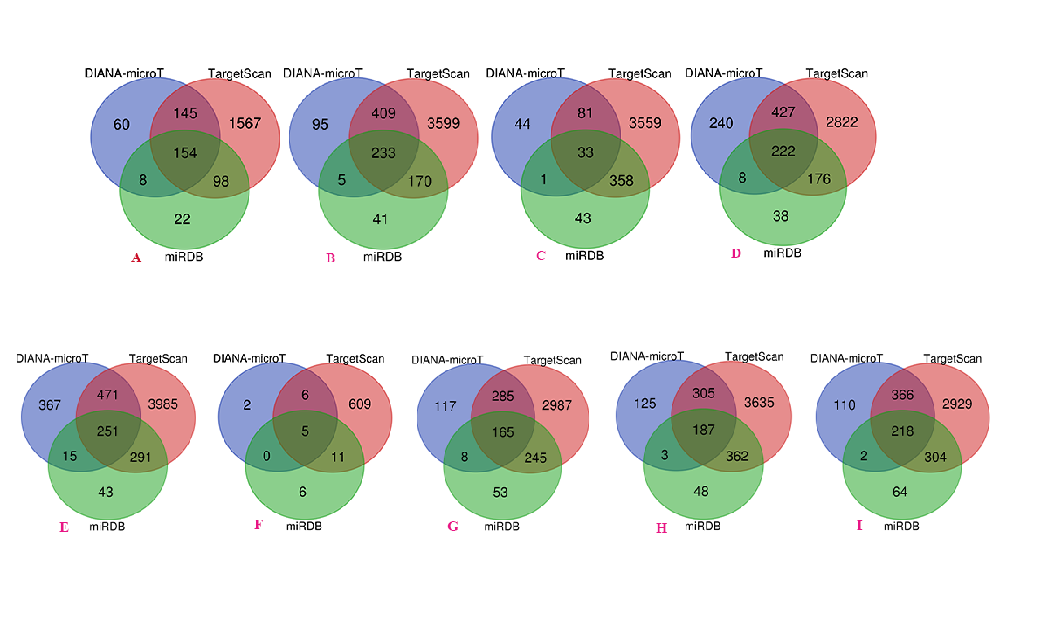

Supplement: Supplementary file 2 [file Image2.tif]
